# Giant nonencapsulated pubic lipoma: a case report and literature review on differential diagnoses of atypical lipomas

**DOI:** 10.1080/23320885.2026.2659394

**Published:** 2026-05-18

**Authors:** Nona Sabeti, Mahdieh Mottaghi, Mehri Sakhdari, Ezzat Hajmollarezaei

**Affiliations:** a Supporting the Family and the Youth of Population Research Core, Department of Obstetrics and Gynecology, Mashhad University of Medical Sciences, Faculty of Medicine, Mashhad, Iran; b Clinical Research Development Unit, Ghaem Hospital, Mashhad University of Medical Sciences, Mashhad, Iran

**Keywords:** Giant lipoma, pubic lipoma, benign, case report

## Abstract

Lipomas have been previously documented in the literature. Here, we present a rare case of a lipoma with exceptionally unusual size, localization, and structure. Due to exceptional presentation, we reviewed the literature for differential diagnoses. A 62-year-old female presented with a history of a slow-growing mass in the mons pubis over the past five years. The patient underwent open surgical excision. Postoperative pathological examination revealed a tumor measuring 210 × 150 × 90 mm and weighing 1,280 g; notably, no capsule formation was observed, and no evidence of malignancy was detected. During a three-year follow-up, no recurrence was observed. The rare localization and unusually large size of the lipoma may represent a component of a broader diagnostic spectrum, including liposarcoma, lipodystrophy, and rare adipose disorders (RADs). In unusual presentations of lipoma, preoperative MRI is recommended to assist in excluding malignancy, defining lesion margins, and assessing involvement of adjacent tissues, thereby minimizing unnecessary resection and improving surgical outcomes.

## Introduction

Lipomas are benign tumors composed of adipose cells, typically surrounded by a thin sheath of fibrous tissue, with a prevalence of 1% and an incidence rate of 2.1 per 1,000 individuals [1]. Lesions exceeding a diameter of 10 cm or a weight of at least 1,000 g are classified as ‘giant’ lipomas [2]. The occurrence of giant lipomas, especially in unusual areas such as the pubic region, is extremely rare.

The etiology of lipoma remains unclear; however, studies have demonstrated that approximately two-thirds of lipomas exhibit genetic abnormalities [1]. In addition, the development of adipose neoplasms may occur post-traumatic as a result of localized proliferation of adipocytes. Further risk factors include obesity, alcohol consumption, liver disease, and glucose intolerance [1,3,4]. Notably, recent large-scale data indicate that the prevalence of metabolic disorders is significantly higher in patients with lipomas compared with controls, including dyslipidemia (83.8% vs. 23.8%), hypertension (64.1% vs. 17.9%), type 2 diabetes mellitus (38.4% vs. 9.0%), and obesity (25.4% vs. 17.7%) [5].

Typically, lipomas are enclosed by a thin, smooth capsule and exhibit characteristics identical to the adjacent subcutaneous fat. Histopathological diagnosis is established by the presence of mature adipocytes, and a surrounding capsule is not a necessary diagnostic criterion. The term “non-encapsulated lipomas” has been used to describe cases with palpable subcutaneous masses that are indistinguishable from the surrounding adipose tissue [6–8].

While lipoma is a well-known benign lesion, the originality of this case report lies in its unprecedented presentation. A 21 cm-diameter, nonencapsulated lipoma localized in the pubic region is exceptionally rare and distinguishes our patient from previously reported cases. This case is reported in line with the SCARE 2025 criteria [9].

## Case presentation

A 62-year-old postmenopausal woman presented to the emergency department with a complaint of a large, painless pubic mass. The mass had gradually increased in size over the past five years, causing difficulty in walking and limiting visualization of the genital region. The patient’s medical history was notable for type 2 diabetes mellitus for ten years, managed with metformin 500 mg twice daily. Additionally, she had hypertension and coronary artery disease for one year, treated with acetylsalicylic acid 80 mg once daily, clopidogrel 75 mg daily, valsartan/amlodipine/hydrochlorothiazide 160/5/12.5 mg once daily, and atorvastatin 40 mg once daily.

**Figure 1.**
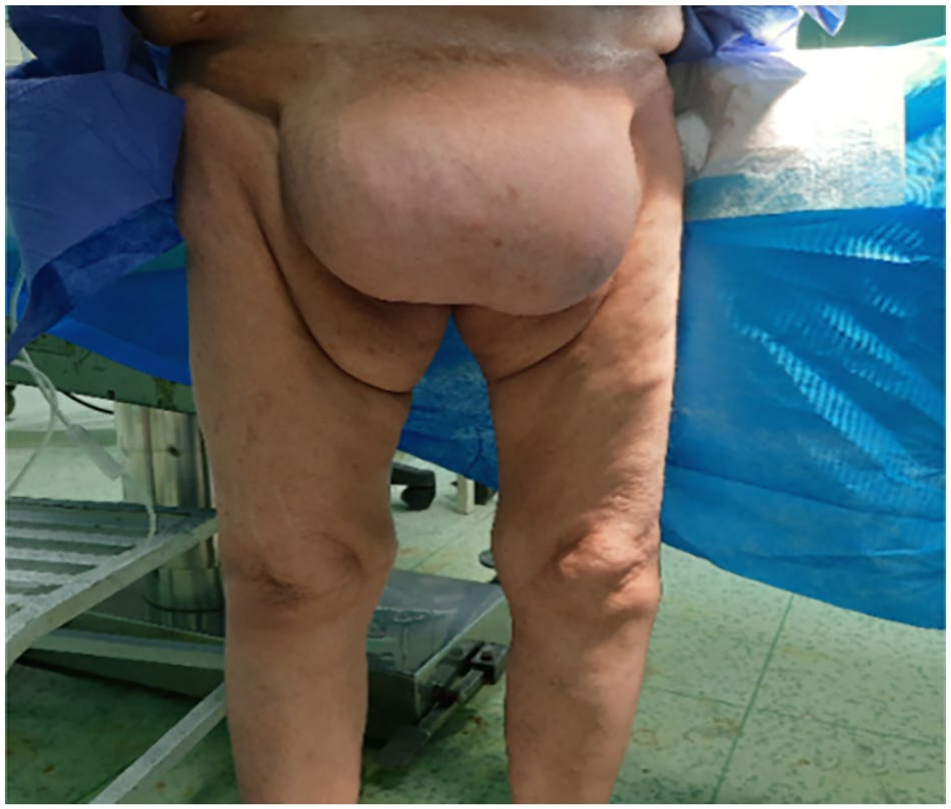
The clinical presentation of giant pubic lipoma.

Upon admission, the patient had a temperature of 37 °C, a pulse rate of 88 beats per minute, and a blood pressure of 130/80 mmHg. Physical examination revealed a well-defined, firm, non-tender mass measuring approximately 12 × 12 cm in the pubic region (Figure 1). Ultrasonography revealed a homogeneous, hyperechoic mass measuring 100 × 120 × 180 mm on the mons pubis, indistinguishable from the subcutaneous adipose tissue of the lower abdomen, suggestive of a giant lipoma. Laboratory tests were within normal range (Table 1).

**Table 1.** Laboratory results upon admission.

	Result	Laboratory Normal Range	Unit
Hemoglobin	12.5	11.9-15	g/dL
Hematocrit	38.5		%
Platelet	195	150-400	×10³ cell/mm³
Red blood cells	4.2	4.2-5.4	×10⁶ cell/µL
Creatinine	1.2	1-1.5	mg/dL
Urea	49	15-45	mg/dL

Preoperative magnetic resonance imaging (MRI) was recommended due to the size of the lesion to exclude the possibility of malignancy; however, the patient declined because of financial limitations. Considering that the mass had been present for five years without evidence of rapid growth or concerning features, the clinical suspicion of malignancy was low, and the surgical team proceeded without performing MRI.

The patient underwent local excision of the lesion under spinal anesthesia. A transverse incision of 8 cm was made over the bulging area of the mass. Upon exposure, the lipoma capsule was not clearly delineated from the underlying tissue. The lesion was completely dissected from the underlying tissue and sent for histopathological examination. After placement of two Nelaton drains to facilitate the drainage of possible hematoma or seroma, the subcutaneous tissue was sutured. The excess skin was excised to enhance the cosmetic outcome of the procedure, and subsequently was closed. Finally, a compressive dressing was applied.

**Figure 2.**
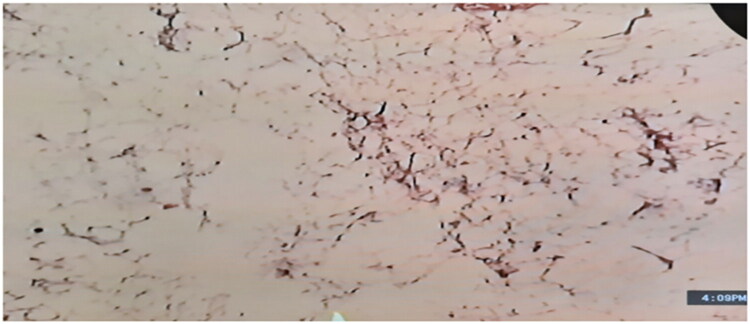
The histological examination of specimen demonstrated mature adipocytes consistent with subcutaneous lipoma (H&E, original magnification 400x).

The gross pathological assessment revealed yellowish white lesion measuring 210 × 150 × 90 mm, weighing 1,280 g with no surrounding defined fibrous capsule. As shown in Figure 2, microscopic pathological evaluation confirmed a benign adipose neoplasm consisting of mature adipocytes, with no evidence of malignancy including cellular atypia, nuclear pleomorphism, or necrosis. There was no infiltration into adjacent soft tissues, and no indications of malignancy were observed. Additionally, no significant inflammatory infiltrate, vascular proliferation, or stromal reaction was noted. The findings are consistent with lipoma.

**Figure 3.**
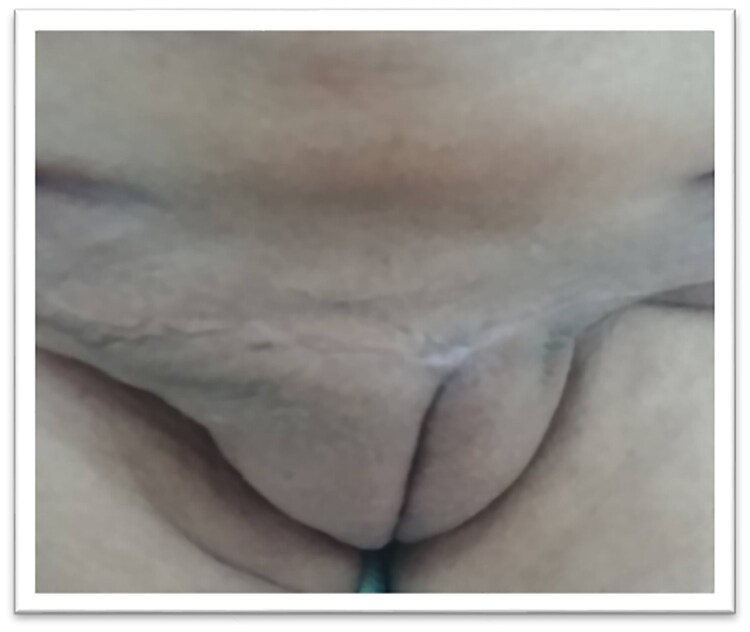
Postoperative image showing the cosmetic result three weeks after surgery.

The postoperative recovery was uneventful, and the patient was discharged on the second postoperative day with vitamin C and Bromelain supplements to facilitate wound healing. Comprehensive postoperative care, including wound management instructions, was provided. Regular follow-up visits were scheduled to monitor for potential recurrence or complications. At three weeks postoperatively, the patient remained asymptomatic, with no clinical evidence of recurrence (Figure 3). At the last follow-up, conducted three years after surgery, there was still no sign of recurrence.

## Discussion

In this case report, we presented a rare occurrence of a giant lipoma in the mons pubis of a healthy-appearing woman with no history of HIV or other medical conditions associated with lipodystrophic diseases. Although previous studies reported cases of vulvar lipoma [10–12], to our knowledge, no similar cases have been previously reported in the available medical literature.

The primary differential diagnosis in our patient was lipoma, confirmed through histopathological evaluation. Similarly, Nazan et al. reported a lipoma of mons pubis in a 48-year-old otherwise healthy woman with no history of HIV or other lipodystrophic conditions which was sized 20 × 15 × 5 cm [13]. While the lipoma in our case was not encapsulated, the previously reported lipoma was enclosed in a fibrous capsule and was smaller in size. Park et al. reported a case of non-encapsulated fibrolipoma in a 32-year-old Korean man in the right lower pubic area measuring 4.0 × 2.5 × 2.5 cm. While the localization of the tumor was similar to our case, the patient was not a case of giant lipoma [14].

Liposarcoma, as the second possible preoperative diagnosis, should have been considered a major concern. However, in this particular case, several features reduced the clinical suspicion of malignancy. The lesion had been present for many years without evidence of rapid growth, sudden enlargement, or associated systemic symptoms.

The third possible diagnosis involved lipodystrophy. Lipodystrophy, also known as fat redistribution syndrome, characterize by a significant lack or loss of subcutaneous adipose tissue; however, increased adipocytes in other areas can lead to misdiagnosis. Lipodystrophies are classified into two categories, hereditary and acquired such as Human immunodeficiency virus (HIV)-associated Lipodystrophy [15]. An observational study in Italy on 582 HIV-infected patients with known lipodystrophy and found that the overall prevalence of pubic lipoma was 9.4% (*p* < 0.0001). They suggest that pubic lipoma might be a presentation of the HIV-associated lipodystrophy syndrome [16].

The fourth differential diagnosis involved rare adipose disorders (RADs) [15]. RADs are often being misdiagnosed with multiple symmetric lipomatosis, lipedema, and Dercum’s disease. RADs increase the accumulation and alter the distribution of the subcutaneous adipose tissue.

While preoperative imaging using high-resolution modalities such as MRI is not necessary prior to surgical interventions for lipomas, it is recommended in cases of giant lipomas due to the risk of malignancy. Additionally, MRI aids in precise marking of lesion margins and assessment of potential involvement of surrounding tissues, minimizing unnecessary tissue resection during surgery. Preoperative marking of the surgical area prevents excessive tissue removal and improve cosmetic outcomes [17,18]. However, in our case, MRI was not performed due to the patient’s financial constraints.

Asymptomatic lipomas are typically managed conservatively. However, in our patient, the lipoma caused difficulty walking. A previous study demonstrated that individuals with pubic lipoma experienced decreased satisfaction with their physical appearance. Notably, the presence of a pubic lipoma was identified as a limitation on normal sexual function [16].

In patients requiring treatment, therapeutic approaches include intralesional injection of sodium deoxycholate, intralesional steroid injection, and a β2-adrenergic receptor agonist, and liposuction may aid in reducing the size of lesion [19,20]. Studies indicated that open surgery remains a superior technique for the removal of giant lipomas compared to liposuction due to decreased risk of recurrence and lower potential damage to surrounding tissues [21]. When surgical excision is indicated, early intervention is recommended, as removing the lesion at a smaller size minimizes the damage of adjacent joints, nerves, and blood vessels, leading to a less invasive surgical procedure. A Nigerian study revealed that individuals with large lipomas often presented late due to economic barriers and surgical anxiety, leading to a more advanced disease at the time of diagnosis [22].

During follow-up period, our patient did not experience recurrence. Lipomas often do not recur following excision. However, to prevent recurrence, it is essential to ensure the complete removal of the surrounding fibrous capsule during surgical excision [1].

This case underscores several unique features. First, to our knowledge, no other cases of non-encapsulated lipoma in the pubic region have been reported. Our literature review revealed that our study was the first documented case of a giant non-encapsulated pubic lipoma. Second, we present a giant lipoma, a condition of particular interest in previous case reports [23]. Third, it highlights the importance of considering liposarcoma, lipodystrophy, and RADs in the differential diagnosis of large pubic masses. However, our study was limited by a lack of para-clinic imaging such as MRI for objective quantifiable definition of pubic lipoma.

## Conclusions

In conclusion, this case highlights an atypical presentation of a giant, non-encapsulated pubic lipoma in an otherwise healthy individual and underscores the importance of considering a broad differential diagnosis, including liposarcoma, lipodystrophy, and RADs. In unusual cases of lipoma, MRI is recommended prior to surgical intervention not only to help rule out malignancy, particularly liposarcoma, but also to accurately define lesion margins and assess involvement of surrounding tissues. This approach minimizes unnecessary tissue removal, especially in non-encapsulated lipomas. Regular follow-up is also recommended to ensure early detection of any potential recurrence.

## Data Availability

The data used to support the findings of this study are available from the corresponding author upon request.

## Declaration of artificial intelligence (AI) use

Generative artificial intelligence (ChatGPT, OpenAI, GPT-4, March 2024 release) was utilized exclusively to enhance the linguistic quality, grammatical precision, and structural coherence of the manuscript during its drafting and revision phases. The AI was not involved in the generation of clinical data analyses, the interpretation of medical images, or the creation of figures. Only anonymized, non-sensitive manuscript text was provided as input, in full compliance with GDPR and HIPAA regulations; no identifiable patient information or imagery was submitted. All AI-influenced content underwent thorough manual review by the authors, who assume full responsibility for the integrity and accuracy of the final manuscript. Examples of AI prompts are available upon reasonable request.
